# Implicit cognition as a moderator of brief motivational interventions for alcohol

**DOI:** 10.1186/1940-0640-7-S1-A46

**Published:** 2012-10-09

**Authors:** Tibor Palfai, Brian Ostafin

**Affiliations:** 1 Department of Psychology, Boston University, Boston, MA, USA; 2 Addictive Behaviors Research Center, University of Washington, Seattle, WA, USA

## 

Hazardous alcohol use is a function of implicit cognition, i.e., nonconscious influences on appetitive behavior stemming from memory associations that are automatically activated upon exposure to alcohol cues. Implicit cognition is typically not addressed by alcohol brief intervention (BI), which targets explicit or conscious processes underlying alcohol restraint (e.g., readiness-to-change). The potential role of implicit cognition in BI outcomes has not been well-studied. Eighty-nine hazardous drinking college students (Alcohol Use Disorders Identification Test [AUDIT] score > 8) participated in a general health study for course credit. Students were randomly assigned to one of two conditions: assessment only or a 10-15 minute motivational intervention. Students completed measures of alcohol use and readiness-to-change at baseline and at 6-week follow-up. In addition, they completed an alcohol-specific implicit association test (a response-time measure of alcohol approach). The motivational intervention resulted in a significant increase in readiness-to-change immediately following the intervention. However, the effect of the intervention on change in quantity of alcohol use per drinking occasion at follow-up was significantly moderated by implicit cognition. Simple slopes analyses showed that those with low implicit appetitive responses showed significantly less drinking when exposed to the intervention condition, while those with high implicit cognition did not. Similarly, readiness-to-change scores predicted declines in drinking for those low in implicit cognition but not for those who showed high levels of implicit cognition. These results suggest that the effect of brief motivational intervention may be less effective among those with strong implicit alcohol-approach associations, and that implicit cognition may be important to address in subsequent BI development.

